# *Nephila edulis*—breeding and care under laboratory conditions

**DOI:** 10.1007/s00427-020-00649-6

**Published:** 2020-01-31

**Authors:** C. Liebsch, M. Fliess, J. W. Kuhbier, P. M. Vogt, S. Strauss

**Affiliations:** Department of Plastic, Aesthetic, Hand and Reconstructive Surgery, Hannover Medical School, Hannover, Germany

**Keywords:** *Nephila edulis*, Keeping, Breeding, Spider silk rearing, Spider silk research

## Abstract

Due to fascinating mechanical and biological characteristics spider silk is of great interest in many research fields. Among the orb-weavers *Nephila edulis* is one of the species used as source for natural spider silk in laboratories. Under appropriate conditions, animals can be kept and bred easily. This manuscript gives information about the spiders’ natural habitat, behavior, and breeding and compares them with the established methods and conditions within a research laboratory. Keeping conditions and methods of rearing are described in detail. Within a keeping facility with reliable supply of food, cannibalism rate is significantly reduced and spiders mate all year long. Cohabitants of the genus *Steatoda* are routinely found in laboratory keeping. While these small spiders do not pose a threat to *Nephila edulis*, cellar spiders (family *Pholcidae*) have to be extracted as they have been observed hunting for *Nephila* spiders.

## Introduction

**Fig. 1:**
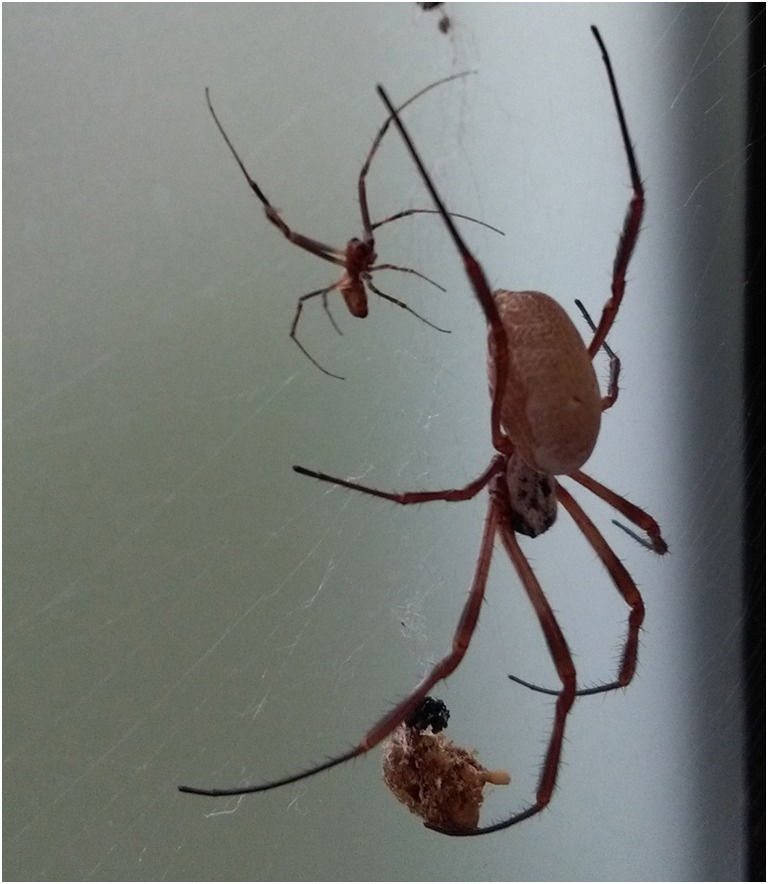
Adult *Nephila edulis*, female on the right, male on the left

The golden silk orb-weaver (*Nephila*) is a long-legged, 2 to 6 cm-sized tropical spider of the order of *Araneae*. The English name refers to its often golden-colored, delicate but stable threat which is used for building webs. Due to fascinating mechanical and biological characteristics, spider silk is of great interest in many research fields, including synthetic production of the material. The species name *Nephila edulis* originates from the word “edible” as the first describer noted that natives of New Caledonia were eating the spiders. While its natural habitat is located in Australia, *Nephila edulis* (Fig. 1) is kept and bred for scientific purposes at the Kerstin Reimers Laboratory (KLR), Department of Plastic, Esthetic, Hand, and Reconstructive Surgery at Hannover Medical School since 2004, successfully. While larger sized species such as *Nephila inaurata madagascariensis*, *N*. *pilipes*, *N*. *maculata*, or *N*. *clavipes* exist, *N*. *edulis* became the “laboratory standard spider” at the KRL due to better breeding results as well as easier care and handling under laboratory conditions.

The medical use of spider silk has already been described in ancient literature e.g., by Pliny the Elder, recommending an application of spider silk on open wounds to promote healing (Newman and Newman 1995). Starting from the search for an absorbable, biocompatible guiding material for regenerating peripheral nerves, spider silk became one of the core themes for regenerative medicine at the KRL. Spiders are kept to collect dragline silk as well as cocoon silk for research on medical applications. With an implant prepared from *Nephila* dragline silk combined with a previously prepared blood vessel, critical size defects of peripheral nerves up to 6 cm have been regenerated successfully in animal models for the first time (Allmeling et al. 2008; Radtke et al. 2011). In course of nerve regeneration, only very mild immune response is observed and spider silk is absorbed completely. Dragline silk was used successfully as wound dressing for wounds up to 9 cm^2^ in sheep (Liebsch et al. 2018) also. Meshes made from the silk show good outcome in a rodent fascia replacement model (Schäfer-Nolte et al. 2014), as suture material (Kuhbier et al. 2011; Hennecke et al. 2013), as scaffold for skin tissue engineering (Wendt et al. 2011) and as supporting matrix for artificial blood vessels (Dastagir et al. 2020). In general, spider silk seems to have good biocompatibility reported by numerous in vitro (Allmeling et al. 2006; Kuhbier et al. 2010; Wendt et al. 2011; Roloff et al. 2014; Kuhbier et al. 2017; Dastagir et al. 2020) and in vivo studies (Allmeling et al. 2008; Radtke et al. 2011; Schäfer-Nolte et al. 2014; Liebsch et al. 2018).

## Habitus

The body length of adult female *N*. *edulis* ranges between 2 and 6 cm while male specimens are usually smaller with around 7 mm, even though males up to 3 cm have been documented.

**Fig. 2:**
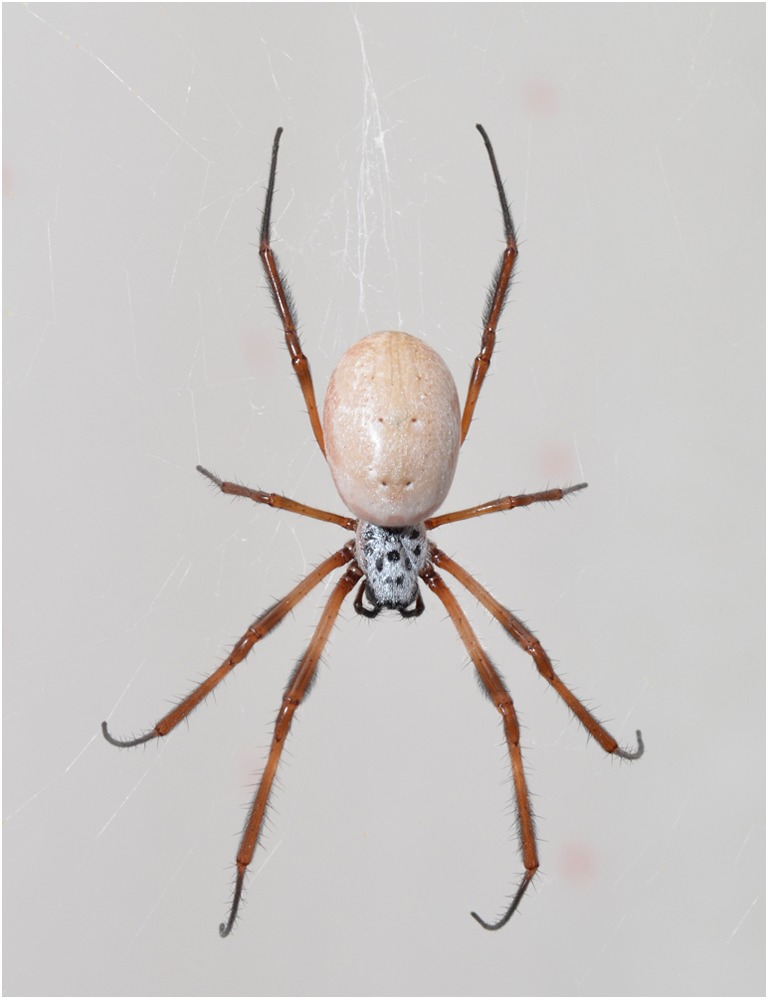
Adult female *N*. *edulis* in typical posture in her web

Eight eyes are located cranially on the first third of the prosoma. Nephila spiders are equipped with pairs of chelicerae and pedipalps and eight legs (Foelix 2011). The female prosoma is dorsally colored in a white and black pattern. The ventral side shows a yellowish pattern. Female *N*. *edulis* have a rounder opisthosoma than other *Nephila* species, which appears brown-silvery. The female’s legs are covered with macroscopically visible tactile hair of dark to bright brown color. The first two pairs of legs are positioned in cranial (anterior) direction, one shorter pair is positioned central and the fourth pair is pointing caudal (posterior direction). On the dorsal opisthosoma, two or three pairs of sigilla are visible. The muscles attached play a role in the process of leg extension as well as in the control of hemolymph pressure. Male spiders have a brown prosoma and opisthosoma accompanied by brown to black legs. Usually, spiders are sitting upside down in their webs (Fig. 2).

## Webs

**Fig. 3:**
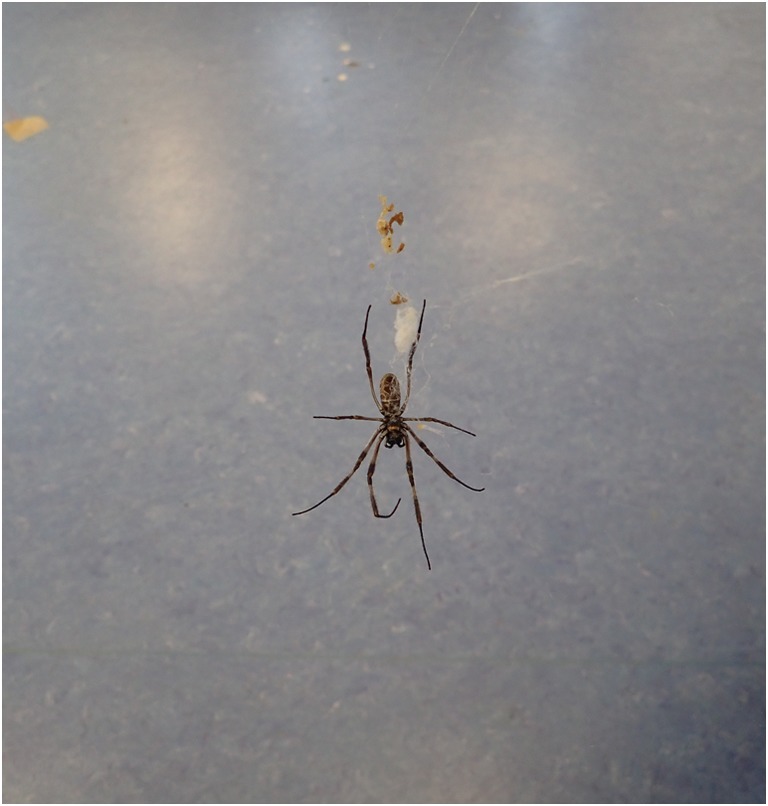
Cross-weaved structures in *N*. *edulis* web at the KRL

Female *N*. *edulis* spiders build large-sized (up to 2 m), asymmetric (Austin and Anderson 1978), and remarkably stable webs, which can even resist storms (Cranford et al. 2012). The spiders spend most of the time within the hub of their web, located in the upper half, while the lower part is sticky for capturing of prey. The capturing strands run pendulum-like in horizontal direction while the auxiliary threads form a spiral. Often small cross-weaved structures can be found above the hub. Literature describes them either as barrier web, which shall protect the spider from predators (Higgins 1992) or as a form of stabiliment (Wiehle 1931), which may also function as sun protection. These structures do also occur in webs of laboratory spiders at the Kerstin Reimers Laboratory but are usually limited in size (max. 1 × 2 cm) and restricted to the position directly above the center of the web (Fig. 3). As these structures excel in collecting water drops from watering at the KLR, they are often frequented by spiders for water uptake. In the native habitat, webs are positioned in locations which are frequented by flying insects for example, between bushes and trees, or close to forests and streams (Harvey 2007). As spiders in the laboratory are fed by hand, animals have adopted to more convenient building of webs in preference of non-exposed places in room corners or near the floor under tables using as many attachment points as possible for the web frame. Only in case of high population density, spiders span their webs across paths used by personnel within the room. If available, spiders prefer to use abandoned old webs rather than building new ones, and damaged webs are patched poorly. These findings match the observations of captive *Nephila clavipes* females (Robinson and Mirick 1971) but are contrary to current knowledge about most of the free living orb weavers. Therefore, it might be an adapted behavior in context of captivity.

## Spinnerets and silk

**Fig. 4:**
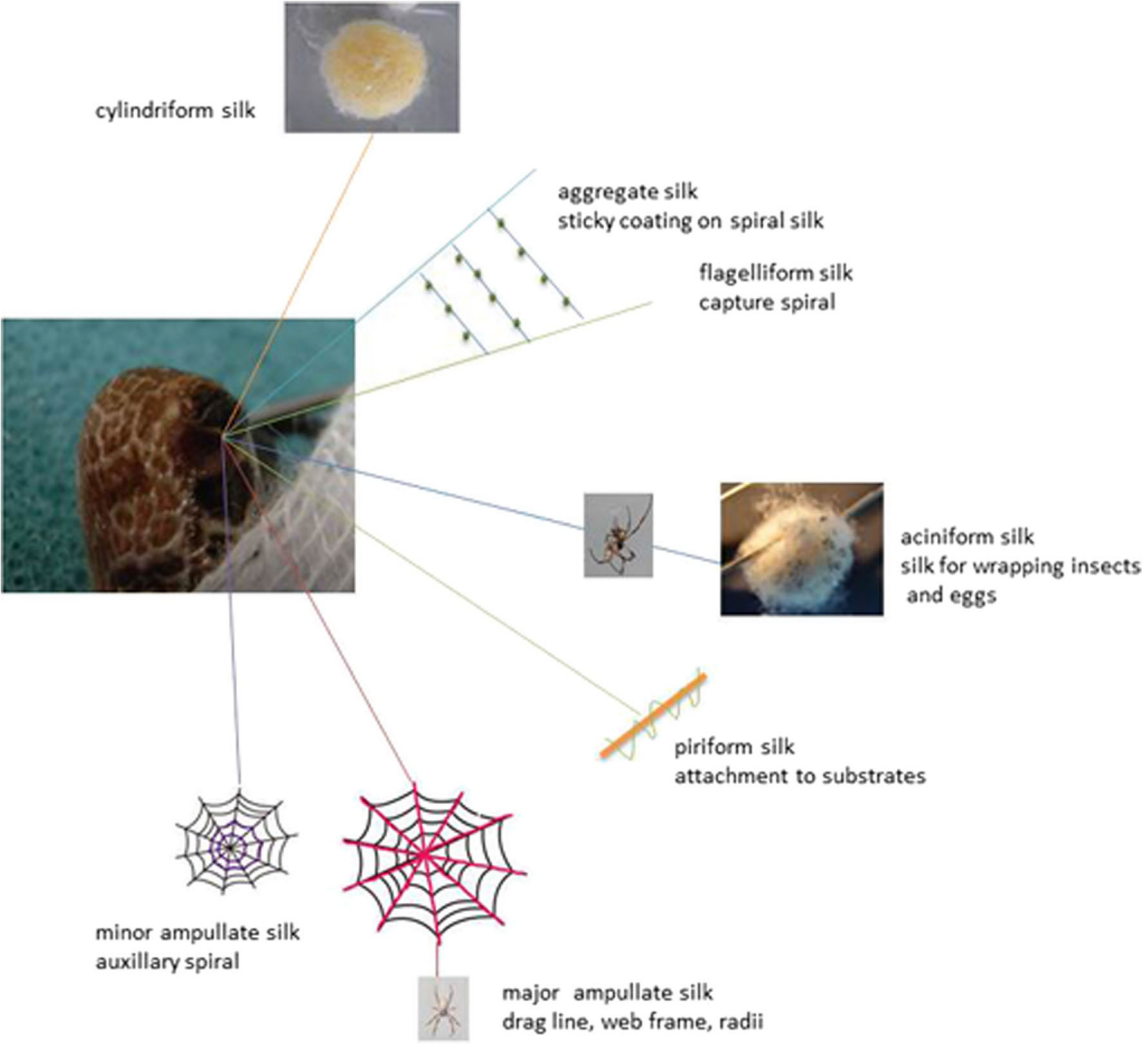
Spinneret and silk types (orientated on Vollrath 2000)

**Fig. 5:**
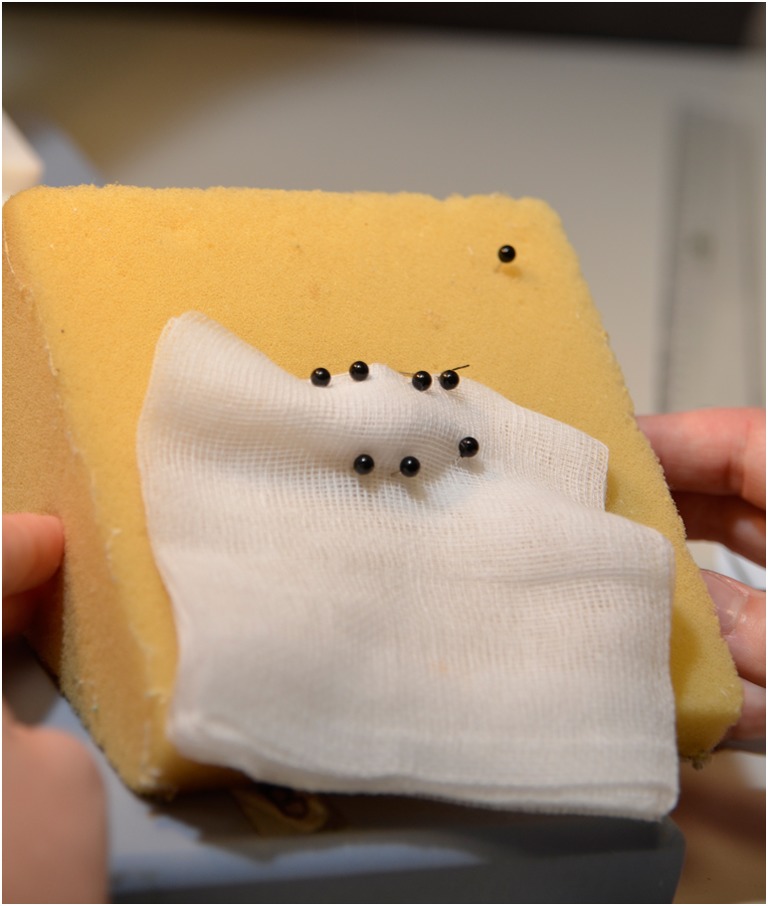
Fixation of a female spider for reeling

**Fig. 6:**
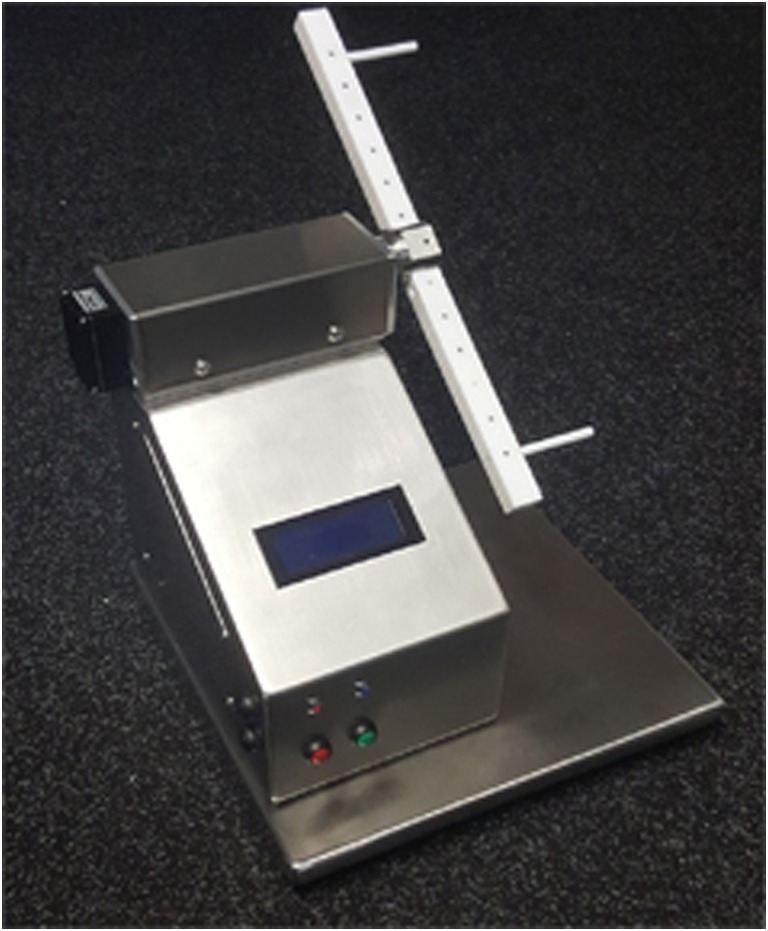
Computerized reeling machine (Feuerhahn and Straub 2017)

The spinnerets are located on the ventral end of the abdomen. Six different types of spinning glands for several types of silk for various applications are described for female spiders (Fig. 4) (Gosline et al. 1986; Vollrath 2000; Lewis 2006): major ampullate for dragline silk and structural components of the web, minor ampullate for the auxiliary spiral of the web, flagelliform for the inner spiral of the web, aggregate for sticky coating of web spiral, piriform for attachment of threats and web, aciniform for packing of food and wrapping of eggs, and cylindrical for capture core threats and outer egg sack. Silk types differ in composition of proteins and therefore also in mechanical characteristics e.g., stability and elasticity (Vollrath 2000). Due to its mechanical and biological characteristics, dragline silk is of major interest for various fields of research. At all times, the dragline connects the spider to the substrate its sitting on and functions as a “safety line.” Gentle pulling of the “safety line” allows collection of silk. During silk extraction, fixation of the spider has proven successful to prevent harm or escape (Fig. 5). Dragline silk can be collected on individual frames which are assembled on a computerized reeling machine (Fig. 6). The reeling machines used at KLR have been developed individually (Feuerhahn and Straub 2017) and are commercially not available. Up to 500 m of silk can be collected from one animal in one reeling process, but the spider will suffer from complete exhaustion. Therefore, silk extraction is generally limited to 100 m twice a week. Afterwards, the spider is placed back in its web, watered, and rewarded (fed) with crickets. Spiders that build a cocoon or are molting should not be used for reeling procedures for a minimum of 3 days.

## Climate

**Fig. 7:**
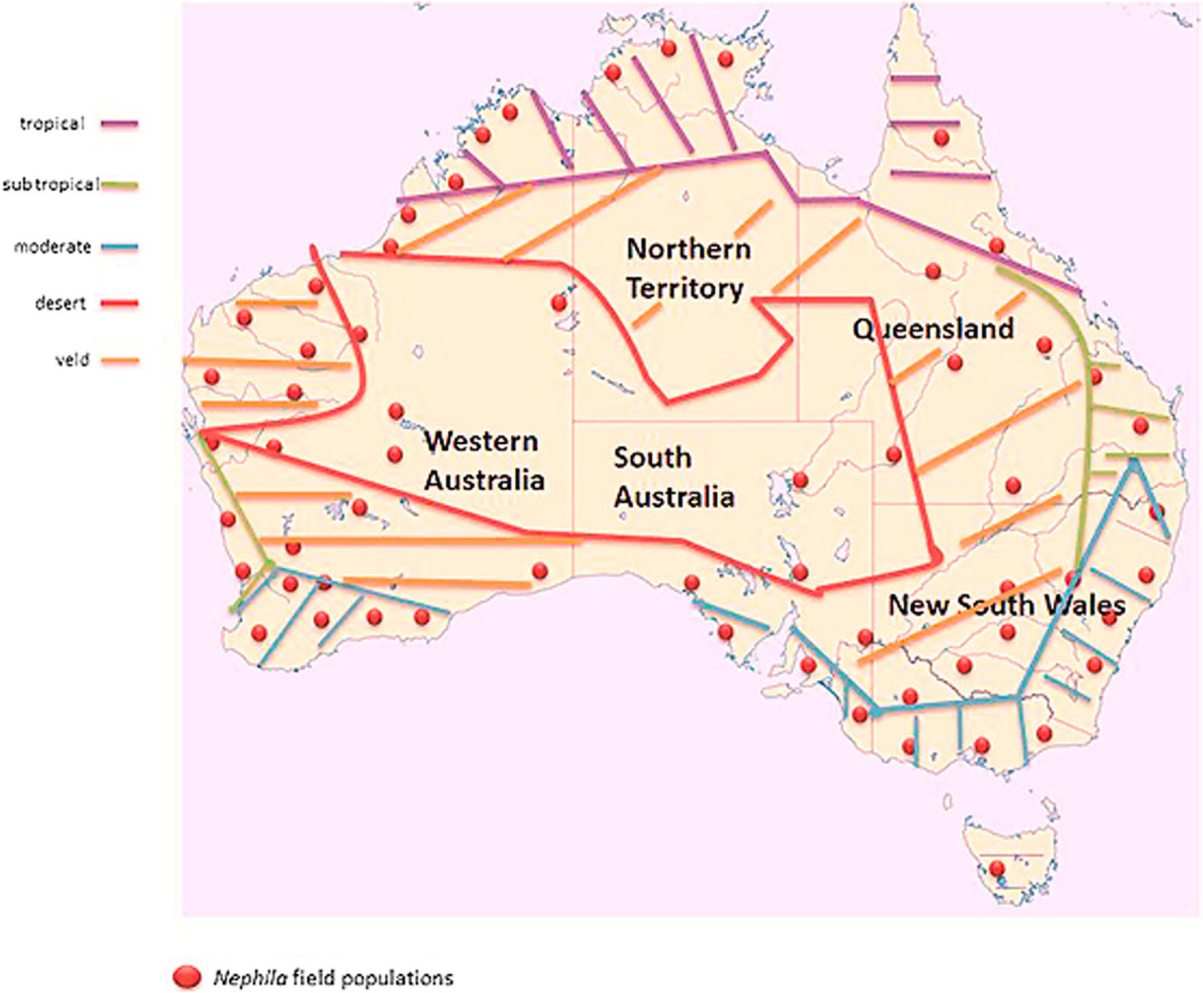
*Nephila edulis* populations and climate zones in Australia

*N*. *edulis* is widespread in Australia within various climate zones of the continent, including the dry center, subalpine, tropical savanna, subtropical, and coastal areas (Fig. 7). Due to their prevalence climatic data for documented field, populations are extremely diverse.

### North

Within the Australian summertime from November to April, strong rainfalls and cyclones occur with a maximum precipitation around 400 mm in January. During this time, temperatures range between 32 by day and approx. 25 °C at night. The other half of the year, from May to October, is dry with around 30 °C by day and 20 °C at night.

### South

The south of Australia is defined by subtropical climate with nearly rain-free summertime at about 30 °C at daytime and winter temperatures around 10 °C, which occasionally drop down to below 0 °C in cold nights.

### West

During summertime, the northern part of west Australia is characterized by strong rainfalls combined with temperatures up to 40 °C. The winters are dry with a day maximum of 29 °C. The southern region is subtropic to temperate and in comparison, dryer than the northern region. Day temperatures reach 31 °C in summer and 12 °C in winter, while night temperatures drop down to 17 °C in summer and 8 °C in winter respectively.

### East

In the east, the climate is tropic to temperate. Strong rainfalls between 1000 and 1500 mm occur during the whole summer time with a temperature between 30 and 35 °C at daytime and 20 to 24 °C at night. The winter is very mild with temperatures ranging from 20 to 25 °C by day and 17 to 9 °C at night.

### Central Australia

The central is very dry and hot with day temperatures exceeding 40 °C in summer. In the mild winter, temperature stays above 20 °C regularly at daytime, dropping down to 4 °C during nighttime (Jones et al. 2009).

### KRL spider room

In the spider room, the temperature is generally above 25 °C with drop downs to 20 °C for short periods during very strong winters. To prevent further drop downs of temperature, the room is equipped with additional mobile heating devices. In summer, temperatures can reach up to 30 °C. Relative humidity is kept in the range from 40 to 60% by day with a tendency to drier climate during summer. From November to March, humidity at night is elevated from 75 to 80%. Vaporizers and programmable timers are used to control the humidity. These are filled with Aqua dest. and cleaned routinely to prevent microbial contamination. Additionally, daily watering of the animals contributes to the indoor climate.

## Predators

There are no detailed studies regarding natural predators of *N*. *edulis*, it seems that this species is only rarely attacked within its web. Tso et al. stated that the coloration and upside down posture protect the animals. Birds, which in part are known to have the ability to sensate colors within the UV spectrum, or bats, seem to qualify as predator. Attacks from these animals on *N*. *edulis* have been observed rarely (Tso et al. 2002). Smaller birds may become entangled in the stable web of the spiders and therefore usually do not feed on them. Only young or sub-adult *N*. *edulis* is hunted. Attacks from wasps have also been observed rarely. The natural enemy of the spider is much smaller in size: larvae of *Anatrachyntis terminella*, a moth, are described to hatch within the cocoons of *N*. *edulis* and to cause mortality up to 60% there (Harvey 2007). Also some species of sphecid or spider wasps hunt for *Nephila* spiders to feed their larvae. Notably, all of these hymenoptera command the skill of UV sensation (Tso et al. 2002).

Under laboratory keeping, most predators have no relevance as the facility is shielded from them. Nevertheless, complete protection is difficult to realize, and sometimes, cellar spiders (family *Pholcidae*) are found inside the room. At KRL, these are removed immediately as they have been observed hunting for *Nephila* spiders.

## Nutrition

**Fig. 8:**
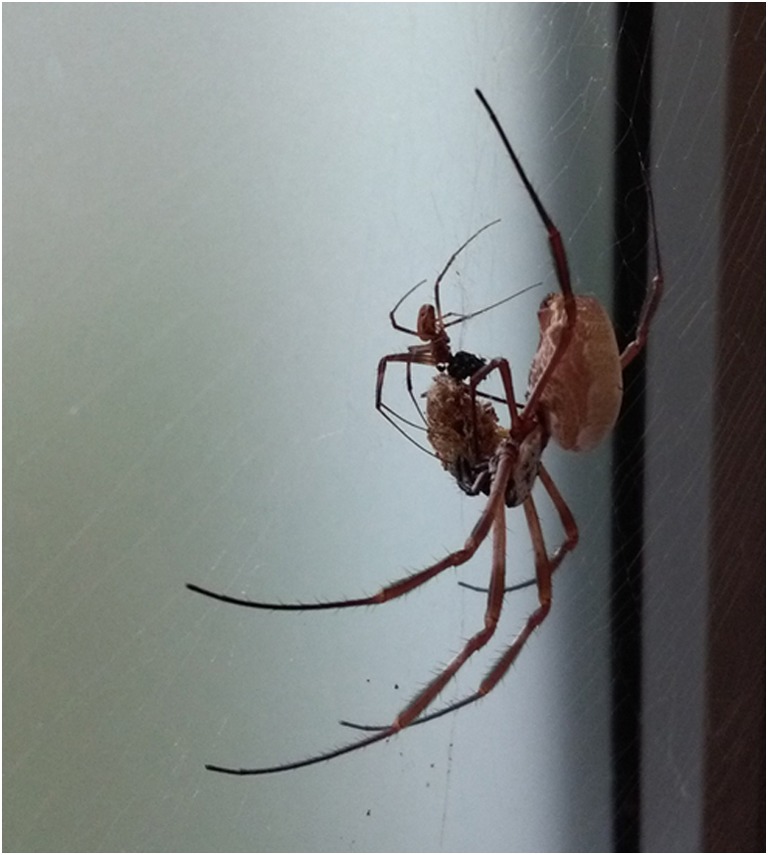
Male feeding on female’s prey

Adult *Nephila edulis* usually feed on insects of appropriate size which are captured in the web. At the laboratory, adult female spiders are fed with adult or semi-adult crickets (*Achaeta domestica*) of appropriate size. As males are not fed, many of them can be observed feeding on prey of female spiders (Fig. 8).

KRL spiders tend to drop prey on the floor instead of wrapping it for storage; thus, crickets are always decapitated freshly to prevent escape of live food from the spider room. Spiders obtain food by hand or by targeted throwing it into their web. The feeding cycle is once or twice a week, with an additional feeding after silk reeling. Watering is performed daily using spray bottles filled with fresh tab water. Webs are carefully moistened as spiders ingest small water droplets.

Crickets are housed in groups in small terrariums within the spider room and fed with chicklet food pellets. Water is available ad libitum in form of agarose gel.

**Fig. 9:**
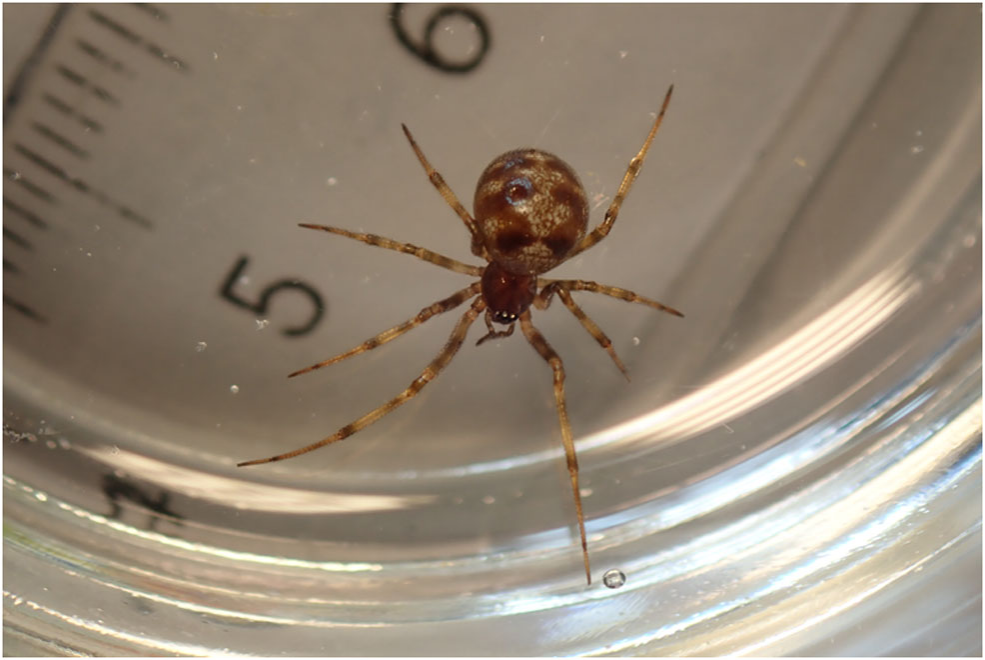
Spider of the genus *Steatoda* collected at the KRL spider room

In the natural habitat, too small prey will be ignored by the web owner but will be fed by smaller kleptoparasitic spiders of the genus *Argyrodes* (Elgar 1989) which live within the web. Interestingly, spiders of the genus *Steatoda* (looks like *Steatoda triangulosa*) are found at the KRL spider room (Fig. 9). These belong to the same family (*Theridiidae*) as the genus *Argyrodes*. As *Steatoda* is also found outside the facility and within the whole building, the spiders most likely immigrated into the keeping room. They are rarely observed directly within *Nephila* webs, but commonly found in narrow gaps or corners in the room, often close to the *Nephila* webs. *Steatoda* spiders in the keeping room seem to feed on escaped flies or small crickets and food debris from *Nephila*.

According to de Crespigny (2001), *Nephila edulis* store as much prey as possible in their webs. The study was performed with wild caught spiders, which were then housed in the laboratory for a short period. Spiders at KRL store only one cricket, if any, and do not try to collect more than the prey they are currently feeding on. Usually, only food debris is stored in the web in form of food cages which are positioned close to the hub. The species is bred since 2004, in the laboratory and therefore might display reduced motivation to store food as fresh prey is regularly available. Although the webs of the laboratory spiders are often quite close to their neighbors, animals usually show no aggression against each other or even cannibalism. This might also be an effect of captivity where food is always available in good quality leading to a reduction of intraspecific competition.

## Molting

As all orb weavers, *Nephila edulis* frequently molts with increasing size. Usually, the animals attach themselves in a “save” place within the web by a molting threat in upside down position. After lateral tearing, the old carapace is lifted and the new drawn out, followed by the legs. After this, the opisthosoma is liberated, too (Foelix 2011). Spiders are extremely vulnerable during this phase until the new exoskeleton has hardened. Therefore, molting and fresh molted spiders should not be handled, watered, or fed.

*N*. *edulis* at KRL do not stop molting after reaching maturity. This is in line with a study of Kuntner (2012), who studied post-maturity molting in *N*. *pilipes*. Therefore, it can be assumed that spiders at KRL show common biological life cycle with their post-maturity molting and production of several cocoons within their lifetime. Females at KRL usually reach an age of 1.5 to 2 years depending on frequency and extent of silk extraction during their lifespan.

## Breeding and rearing

At KRL, no targeted mating is performed. As all adult animals live free in one room, males decide self-initiated which females are frequented (Fig. 1). Approaching a female’s web takes place very carefully and slowly, accompanied by pulling on web threats. This behavior and the copulation have been described before by Uhl and Vollrath (1998). In case of aggressive response by the female, the male remains immobile for several minutes before starting a new approach. If the female still shows aggressive behavior, most of the male spiders migrate to the next web and start a new attempt there. Often males can be observed drawing near while the female is fed which is in line with observations of Uhl and Vollrath (1998) also. According to the study of Uhl and Vollrath (1998), pre-copulatory cannibalism is quite low at 1%, but post-copulatory is notable at 12.2% under laboratory conditions. As the act of copulation is only rarely observable (due to limited on-site time of staff), no data regarding this is available from KRL spider keeping. Dead males have not been found in female webs so far.

**Fig. 10:**
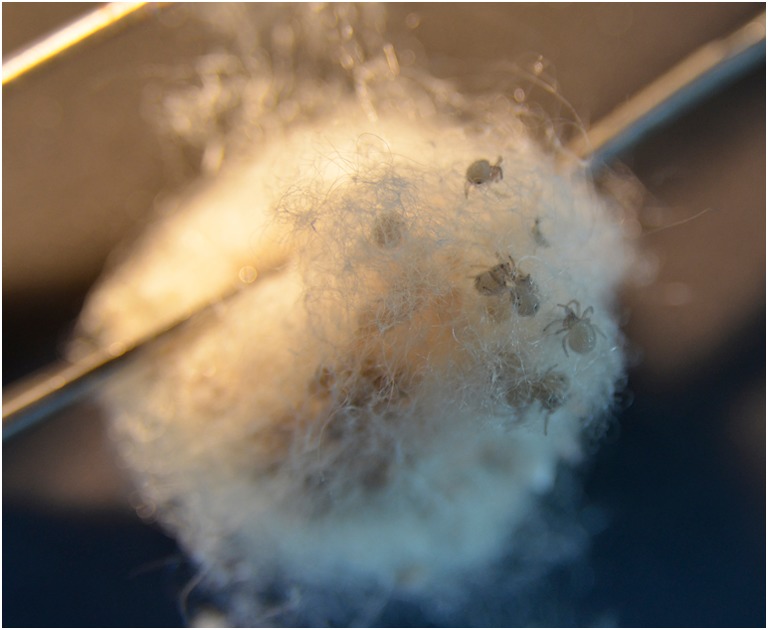
Cocoon with spiderlings

According to Austin and Anderson (1978) *N*. *edulis* mate between February and May, while spiders in laboratory keeping mate all over the year. Cocoons are built frequently also. Cocoons can contain up to several hundred eggs. It takes between 29 and 87 days until spiderlings hatch (Austin and Anderson 1978). Cocoons at KRL are built in “safe” places (from the spider’s point of view) which are located in corners, any kind of ridges, undersides of tables, in lamps, and even in smoke detectors (which can become a serious problem as it can induce nuisance alarms). After completing the cocoon, most spiders stay close to it for a few days. Cocoons are collected when the mother animal has left it and stored in perforated plastic containers at around 25 °C in the keeping room. After hatching, cocoons with spiderlings (Fig. 10) are transferred to a clean-vented container or small terrarium. The containers are equipped with pine bark substrate and small branches on which cocoons can be deposited. It is important to make sure that *Drosophila melanogaster*, which are the first food for the small spiderlings, cannot escape from the terrarium. To prevent a mass escape of *Drosophila*, flightless animals should be used. Usually, young spiderlings share the “large” prey with their siblings and should be left with their cocoon or the first collective web until they leave it self-initiated. To prevent cannibalism of siblings, enough food needs to be available all the time until the third molting. Containers with spiderlings are watered once a day using a spray bottle to elevate humidity. The containers should be just moist, not wet as this would promote mold formation. Spiderlings or cocoons should not be directly sprayed on.

Reaching a size of 0.5 to 1 cm groups of young spiders are placed in bigger terrariums which are also equipped with pine bark substrate and small branches. Spiders of this size are fed with blowflies (*Lucilia sericata*). Terrariums should be cleaned regularly with warm water and a mild detergent and controlled twice a week for mold formation or food debris. Male spiders are released into the room as soon as sex can be determined macroscopically. Usually, animals are placed on a plant or a wall next to a female’s web. From there, males go their way self-initiated.

Reaching a size of 3.5 cm, female spiders are released into the room. Usually, animals are placed in an abandoned old web or close to a place where a new web can easily be built. Many spiders do not migrate long distances. In case a migration of longer distance occurs, stocking density within the closer surrounding might be too high, illumination too slight, and/or the place too exposed. The released females are fed with decapitated adult or semi-adult crickets (*Acheta domesticus*) of appropriate size. As the spiders tend to drop down surplus food instead of storing it, this technique prevents escape of live food from the spider room. Spiders obtain food by hand or by targeted throwing it into the webs.

Due to large amounts of food debris and spider feces on the floor, the room has to be cleaned once to twice a week by moist wiping. As spiders might react adverse on organic solvents or synthetic supplements, only warm water with curd soap is used for cleaning.

## Room equipment

**Fig. 11:**
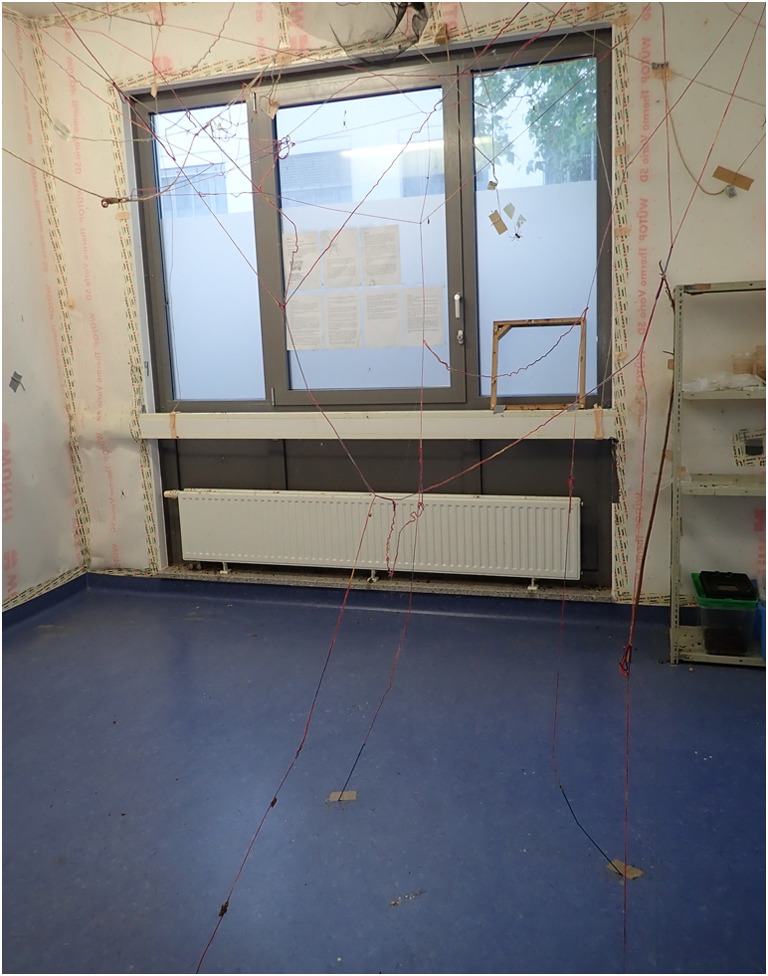
Spider room at KRL with damp-proof interior, vapor retarding foil at the walls, and threads from floor to ceiling

**Fig. 12:**
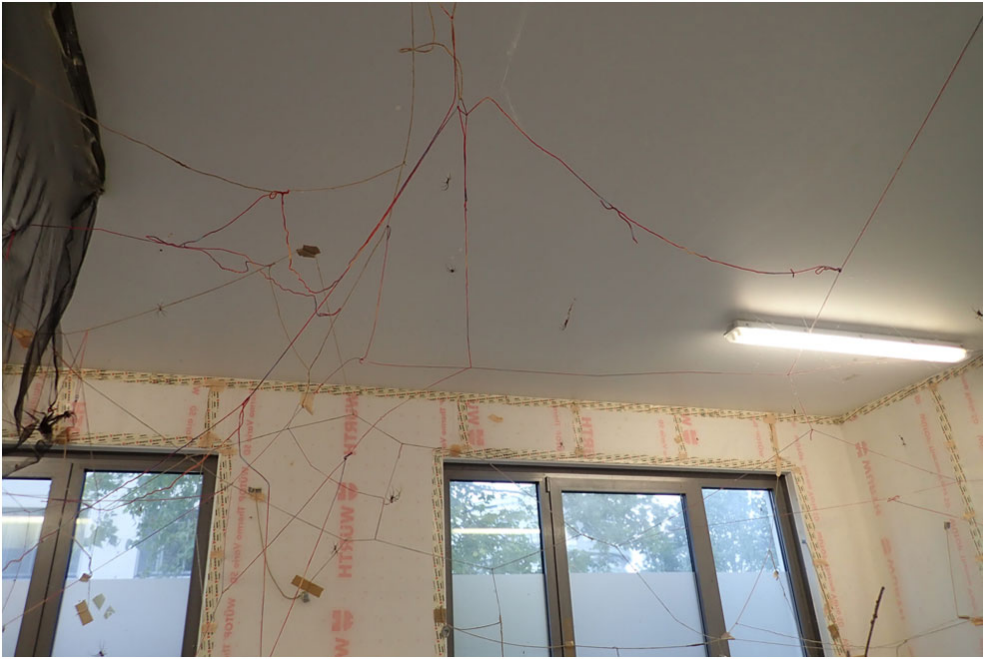
Threads under the ceiling and damp-proof ceiling light

The keeping room should be equipped with damp-proof equipment as relative humidity is temporary very high, and the floor is frequently wet from watering. Protection against electric shock is essential in such surroundings. Ceiling lamps, wiring, and sockets have to be damp-proof. At KRL, the walls are covered with vapor retarding foil (WÜTOP Thermo Vario SD, Würth, Künzelsau-Gaisbach, Germany) (Figs. 11 and 12). The textile lining is accepted excellently by the spiders for attachment of webs. Another important feature in context of safety is the ladder used for animal care. Due to danger of slipping on wet floor or stairs, a stable model with broad stairs and handrail is used.

As *Nephila edulis* is heliophilous, bright illumination should be offered. At KRL, two sites of the room have large windows which allow natural incidence of sunlight. Ceiling lamps are used for illumination on cloudy days for 8 to 10 h. Furthermore, in winter lamps with daylight spectrum and automatic time control are used for additional illumination as otherwise, all spiders migrate close to the windows.

**Fig. 13:**
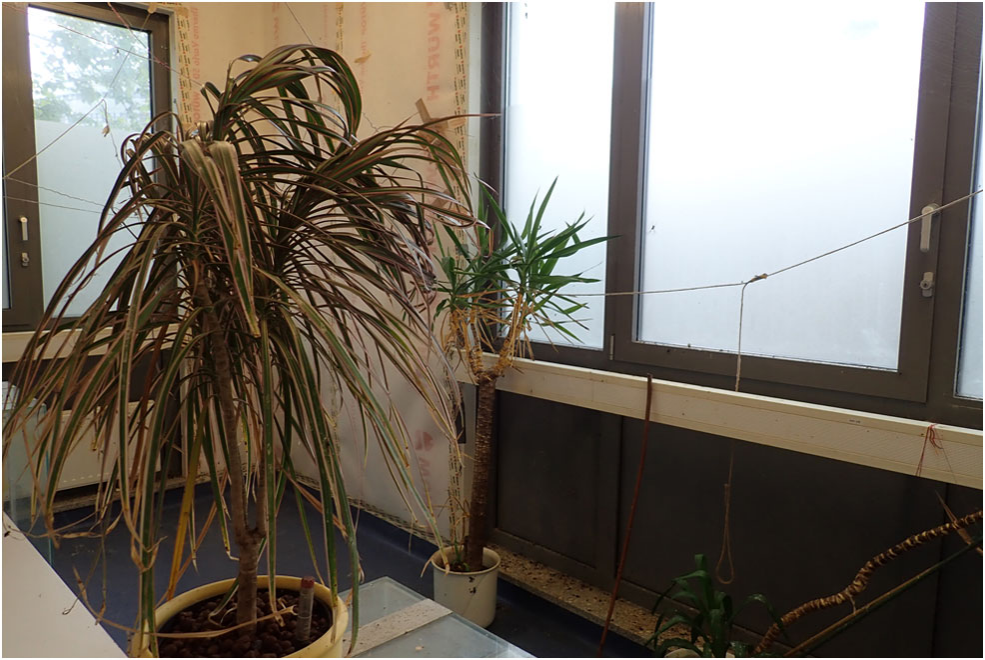
Plants in the spider room

For enlargement of available building space for webs, plants or tree branches are placed in the room (Fig.13). Beside these, small ropes from hemp or wool are spanned across the room and from the ceiling to the floor (Figs. 11 and 12). The ropes are used for attachment of webs as well as for migration of spiders.

To prevent contamination of the whole department with escaped live food or invasion of animals in the keeping room, adhesive traps are installed at the door. Additionally, fly screens are installed in front of the windows and the door.

## Conclusion

*Nephila edulis* can easily be kept, bred, and reared under laboratory conditions. Within years of captivity, since 2004, the species seems to display some adaption e.g., limited to no storage of prey and reduced repair of webs. Cannibalism occurs only rarely as long as enough food is available. If adult spiders are housed free within a room, the interior has to be damp-proof. Animal escapes from, or invasion in the room can be limited by simple preventive measures like installation of fly screens.
